# Virulence Attributes and Host Response Assays for Determining Pathogenic Potential of *Pseudomonas* Strains Used in Biotechnology

**DOI:** 10.1371/journal.pone.0143604

**Published:** 2015-11-30

**Authors:** Azam F. Tayabali, Gordon Coleman, Kathy C. Nguyen

**Affiliations:** Biotechnology Laboratory, Environmental Health Science and Research Bureau, Healthy Environments and Consumer Safety Branch, Environmental Health Centre, Health Canada, Ottawa, Ontario, Canada; University of North Dakota, UNITED STATES

## Abstract

*Pseudomonas* species are opportunistically pathogenic to humans, yet closely related species are used in biotechnology applications. In order to screen for the pathogenic potential of strains considered for biotechnology applications, several *Pseudomonas* strains (*P*.*aeruginosa* (Pa), *P*.*fluorescens* (Pf), *P*.*putida* (Pp), *P*.*stutzeri* (Ps)) were compared using functional virulence and toxicity assays. Most Pa strains and Ps grew at temperatures between 28°C and 42°C. However, Pf and Pp strains were the most antibiotic resistant, with ciprofloxacin and colistin being the most effective of those tested. No strain was haemolytic on sheep blood agar. Almost all Pa, but not other test strains, produced a pyocyanin-like chromophore, and caused cytotoxicity towards cultured human HT29 cells. Murine endotracheal exposures indicated that the laboratory reference strain, PAO1, was most persistent in the lungs. Only Pa strains induced pro-inflammatory and inflammatory responses, as measured by elevated cytokines and pulmonary Gr-1 -positive cells. Serum amyloid A was elevated at ≥ 48 h post-exposure by only some Pa strains. No relationship was observed between strains and levels of peripheral leukocytes. The species designation or isolation source may not accurately reflect pathogenic potential, since the clinical strain Pa10752 was relatively nonvirulent, but the industrial strain Pa31480 showed comparable virulence to PAO1. Functional assays involving microbial growth, cytotoxicity and murine immunological responses may be most useful for identifying problematic *Pseudomonas* strains being considered for biotechnology applications.

## Introduction

*Pseudomonas* species are metabolically diverse and as such, can be found ubiquitously in the environment [1]. The metabolic versatility of *Pseudomonas* species has also generated interest in their industrial use for remediation and detoxification of hazardous environmental pollutants, such as metals, pesticides and phenolic compounds [2].

However some species, such as *Pseudomonas aeruginosa*, are known to cause infections in cystic fibrosis and immunocompromised patients, as well as immunocompetent hosts [3–8]. Infections caused by other species such as *P*. *fluorescens*, *P*. *putida* and *P*. *stutzeri* [9–19] have also been described, albeit with lower frequency.

The advantages associated with industrial and environmental applications involving *Pseudomonas* species need to be balanced with the potential for risk of human health effects. One aspect of this stewardship is timely hazard assessment of individual strains and oversight of their intended use in biotechnology applications to limit environmental and commercial dissemination of potentially pathogenic strains.

To address the human risk aspects of this concern, our efforts have focussed on identifying assays that are capable of distinguishing microorganisms based on their pathogenicity potential [20–22]. Although, several *Pseudomonas*-specific virulence determinants have been identified [23,24], and attributes such as antibiotic resistance, quorum-sensing and biofilm formation greatly enhance the ability of the organism to endure harsh environments and cause infection [25–28], the exact combination of virulence determinants necessary for a pathogenic phenotype remains unclear. Therefore, there is a need for a set of non-specific, versatile, and accurate assays that can be applied to any *Pseudomonas* species, and even extended to any bacterial strain considered for biotechnology applications. The objective of this study is to assess the potential of several biotechnology-related *Pseudomonas* strains to be pathogenic to humans based on a set of functional virulence assays. Similar strategies have been demonstrated using *in vitro* methods for *Acinetobacter* and *Bacillus* species, as well as model of murine innate immune response to distinguish relatively safe from potentially harmful strains [21,22,29].

The study presented here extends our current understanding of the comparative virulence capacity of various *Pseudomonas* strains, and the early toxicological responses of cultured mammalian cells and mice. A prediction of the potential pathogenicity of various *Pseudomonas* species was generated from the cumulative evidence provided through these multiple endpoint assays.

## Materials and Methods

### Preparation of Bacteria

Most *Pseudomonas* strains were obtained from the American Type Culture Collection (ATCC) (Rockville, MD). Their isolation sources and applications are described in Table 1. Comparisons were made to the PAO1 laboratory reference strain that was obtained from the *Pseudomonas* Genetic Stock Center (Dept. of Microbiology and Immunology, East Carolina School of Medicine, Greenville, NC). Pa10752 was selected since it is considered a type strain. Bacterial stocks were prepared from cells grown overnight in trypticase soy broth (TSB). To do this, cultures were washed of soluble material (culture supernatant and debris) with three centrifugation and resuspension steps using sterile phosphate-buffered saline (PBS). Washed cultures were adjusted to 10^8^ colony forming units (cfu) mL^-1^ and frozen at -20°C in aliquots with fresh TSB and 10% glycerol. Viability of frozen stocks was evaluated routinely by spread-plating and colony enumeration.

**Table 1:** Pseudomonas Strain Information Provided by American Type Culture Collection.

Bacterium (other designations)	ATCC #	Biosafety Level	Isolation Source	Application
**PAO1**	15692	2	Burn/wound from patient,Melbourne, Australia	Laboratory reference strain
**P.aeruginosa (ATCC 14138, NCTC 8028, NRRL B-1069, R. Hugh 151)**	10752	2	Haemorrhagic bullae and ulcers	Opportunistic pathogen research
**P. aeruginosa (SGRR2)**	31480	2	Mutant derived from ATCC#31479 (Soil, Salem, Virginia)	(1) Removes oleaginous material from wastewater (2) Opportunistic pathogen research
**P. aeruginosa (Purple Sulfur)**	700370	2	Soil	Reduces nitric oxide
**P. aeruginosa (KC3)**	700371	2	Information not available	No information
**P. fluorescens (Type Strain; NCTC 10038)**	13525	1	Pre-filter tanks, England	(1) Assay of antimicrobial preservatives (2) Reference material for antimicrobial testing (3) Water testing
**P. putida (Type Strain)**	12633	1	Information not available	(1) Catabolizes mandelate (2) Degrades aromatic acids (3) Hydrolyzes d,L-alpha-amino acid amides (4) Metabolizes 2-ketogluconate 2-ketogluconic acid (5) Metabolizes beta-ketoadipate (6) Bacteriophage host
**P. putida (3P; Formally classified as P. fluorescens)**	31483	1	Wastewater lagoon, SouthCarolina	(1) Degrades detergents (2) Degrades surface active agents (3) Removal of surface active agents and detergents from wasterwater
**P. putida (CB 173)**	31800	1	Wastewater from textile chemical plant, Welford, SC	Degradation of phenolics in wastewater
**P. putida (HC 7219)**	700369	1	Soil	Degrades phenol
**P. stutzeri (220)**	17587	1	Bile	No information

### Growth Kinetics

To monitor bacterial proliferation in TSB, sheep serum (sheep blood (Oxoid Co., ON) centrifuged at 4000 x g for 12 min), or fetal bovine serum (FBS) (Life Technologies, Burlington, ON), 10 cfu mL^-1^ of each *Pseudomonas* strain was added into each well (200 μL final volume) of a 96-well plate. Automated turbidity measurements at an optical density of 500 nm (OD_500_) were collected with a multi-well spectrophotometer (Spectromax Plus 384, Molecular Devices Corp.) pre-set to 28, 32, 37 and 42°C to take measurements every 15 min over a 24 h period. Data was visualized with Softmax^TM^ software (Molecular Devices Corp.) and analysed using Microsoft Excel^TM^. Culture wells were routinely monitored by microscopy. Those wells demonstrating turbidity changes with no apparent microbial growth were noted.

#### Antibiotic Minimal Inhibitory Concentration (MIC) Assay

The MIC assay was done as described in Seligy and Rancourt [30]. Briefly, antibiotics purchased from the Sigma Chemical Company, were selected to target different bacterial systems. These were aztreonam, ceftazidime, ciprofloxacin, colistin, doxycycline, gentamicin, and meropenem. The antibiotics were diluted with TSB into each well of a 96-well plate to a final concentration of either 0, 0.38 0.75, 1.5, 3, 6, 12 and 24 μg ml^-1^. Plates were incubated at 37°C for 24 h. For Pf13525, Pp12633, and Pp31800, plates were incubated at 28°C since no growth was observed at 37°C. Following antibiotic incubation, 3-[4,5-dimethylthiazol-2yl]-2,5-diphenyl tetrazolium bromide (MTT) (Sigma Chemical Co.) was added at a final concentration of one mg mL^-1^. Plates were then incubated for another two hours for bioreduction by viable bacteria to MTT formazan. The MIC value was defined as the minimum value for which no bioreduction of MTT into formazan was observed. The MICs from four to six separate experiments were used to determine a classification of sensitive, intermediate or resistant according breakpoints set by the European Committee on Antimicrobial Susceptibility Testing (EUCAST). Each bacterial strain was then assigned an score, termed the SIR score, based on the total number of susceptible (value of 0), intermediate (value of 1), and resistant (value of 2) breakpoints observed. The greatest SIR scores revealed the most resistant strains and the most ineffective antibiotics.

### Toxin Production

The production of a hemolysin by *Pseudomonas* strains was evaluated as described in a previous publication [21]. Briefly, sterile, defibrinated sheep blood (Cedarlane Laboratories, Hornby, Ontario) was washed three times with PBS, before mixing at a final concentration of 5% (v/v) with 1.25% (w/v) autoclaved agar cooled to 55°C, containing sheep blood base (1.4% (w/v) pancreatic digest of casein, 0.5% (w/v) sodium chloride, 0.45% (w/v) peptone, 0.45% (w/v) yeast extract). After agar solidification, each *Pseudomonas* strain was deposited (10^3^ cfu in 10μL) onto the surface of the plates, and incubated at 28°C or 37°C for up to 6 days. Plates were monitored daily for progression of haemolysis.

Production of pyocyanin and pyoverdine–like substances were monitored on solid-phase cultures. The *Pseudomonas* strains were inoculated on various culture media (MacConkey, nutrient broth, TSB, starch, or urea agars (Oxoid Co.) at 28°C or 37°C. At 24, 48 and 168 h after inoculation, colonies were evaluated for blue-green pigmentation, which is typical of pyocyanin, or fluorescence (using a hand-held ultraviolet A light source), which is typical of pyoverdine [31–33].

### Mammalian Cell Culture and Exposure Effects

Human HT29 colonic epithelial cells (HTB-38) were obtained from the ATCC. Cells were maintained in Dulbecco’s Modified Eagle’s Medium (DMEM) with high glucose (25 mM), supplemented with 10% FBS +/- 50 μg mL^-1^ gentamicin [21,29].

Cytotoxicity of the *Pseudomonas* strains towards mammalian cells was monitored as described previously [21,29]. HT29 cells were exposed to 10^6^ cfu for 24 h in 96-well cell culture plates. Following exposure, MTT was added to the wells at a final concentration of one mg mL^-1^. The plates were incubated for two hours at 37°C. The wells were rinsed twice with PBS to remove non-adherent cells and bacteria. Following addition of 100 μL of dimethyl sulfoxide (DMSO) (Sigma) in each well, solubilized-formazan coloration was measured at OD_505_. The metabolism of MTT into its formazan product was referred to as bioreduction activity, and was expressed as percentage activity compared to that of PBS-treated cells.

### Animal Exposures

Animal exposures were done according to a previous publication [22]. Female Balb/c mice were purchased from Charles River Laboratories Inc. (Saint-Constant, Quebec) and were maintained and treated in a pathogen-free biosafety level 2 containment facility. Only animals between 18 and 23g (6 to 8 weeks) that had been acclimatized for at least one week were used for experiments. Mice had access to food and sterile water *ad libitum*. Mice were endotracheally instilled with bacteria by first lightly anesthetizing them with isoflourane, and then aerosolising a dose of 10^6^ cfu suspended in 25 μL of saline directly into the lungs with a Microsprayer^TM^ (Penn Century, Philadelphia, PA). Mice were monitored to ensure that they recovered from the procedure within two minutes. All procedures involving animals were approved by the Health Canada Animal Care Committee and overseen by a board-certified veterinarian.

### Blood Collection, Haematology and Plasma Analysis

Both bacteria- and saline-treated mice were anaesthetized and approximately 500 μL of blood was collected by cardiac puncture into blood collection tubes containing ethylenediaminetetraacetic acid (EDTA). Tubes were gently mixed to prevent clotting. An aliquot of the blood was diluted 1:1 with Beckmann-Coulter diluent and analysed with a Beckmann-Coulter Ac^.^T 5-Diff^TM^ Haematology Analyser. The analyser was used to measure levels neutrophils, lymphocytes, monocytes, eosinophils and basophils. The remainder of the blood was centrifuged at 1000 x g for 10 min to collect plasma, which was stored at -80°C until use. Plasma samples were used with ELISA kits for measurement of serum amyloid A (SAA) (Biosource International Inc., Camarillo, CA).

### Lung Tissue Analysis

To examine clearance of the bacteria from the lungs, at various times post-exposure, three to four animals per treatment per time-point were euthanized at 2, 4, 24, 48, 96 and 168 h with isoflurane followed by cervical dislocation. Lungs were excised and then liquefied in one mL of saline with a hand-held homogenizer (PowerGen 125, Fisher Scientific). Serial dilutions of the lung homogenate were plated on Luria-Bertani -agar plates and cfu content was determined after 18 h at 37°C.

Granulocyte infiltration into tissues was monitored by microscopy using antibodies to granulocytes (anti-Gr-1). Lung pieces were placed into 4% (w/v) paraformaldehyde for 18–24 h, followed by storage in 70% ethanol at room temperature until further processing. Tissues were sectioned with a vibrating microtome (Vibratome Inc., St. Louis, MO). Sections (25–60 μm) were incubated for 60 min in blocking/incubation buffer (0.05% Tween-20), 90 min at 37°C in 1:50 primary antibody (eBioscience, San Diego, CA) and 60 min with 1:200 TRITC-conjugated anti-rat IgG. Two PBS rinses were done between each step. Sections were mounted with Prolong^TM^ antifade solution (Molecular Probes, Eugene, OR) and viewed with a Nikon TE2000 inverted microscope with epifluorescence optics, connected to a Nikon C1 Laser Scanning Confocal unit with three lasers (488nm, 543nm, 633nm).

The levels of IL-1β, IL-6 and TNF-α in the lungs were measured as described by us previously [22]. Portions of lung were lysed and homogenised with bead-based system (PowerLyzer 24; MoBio Laboratories Inc., Carlsbad, CA). Simultaneous measurement of cytokines was done with pre-conjugated bead kits (BioRad, Mississauga, ON) using a multiplex bead array system (Bioplex 100 or 200, BioRad). Data analysis was done using the instrument software and Microsoft Excel.

### Data and Statistical Analysis

Data was analysed and graphed using Microsoft Excel^TM^ software. The significance of the differences between treatments was determined by analysis of variance (ANOVA) followed by a post-hoc Dunnett’s Multiple Comparison Test (Dunnett, 1980). Statistical analyses were done with Sigmaplot version 11.

## Results

### *Pseudomonas* Strains

Most strains used in this study are either being considered, or currently being used in biotechnology applications. They were selected since they are detailed on the Domestic Substances List of the Canadian Environmental Protection Act (1999), which means they are or were present in Canada for biotechnology applications at relatively high levels. Their isolation sources and applications are described in Table 1. The bacteria were isolated from clinical, environmental, industrial or unknown sources.

### Bacterial Virulence Attributes

The capacity to grow at various temperatures was tested by monitoring OD_500_ in different media. Table 2 summarises these results. All strains were able to grow at 28°C in TSB medium. Pf13525 and Pp31800 were slow to proliferate at 32°C in TSB, and did not proliferate at higher test temperatures. All Pa strains were able to grow at 37°C in TSB, but only some Pa strains were able to grow slowly at 42°C. Notably, Pf13525, Pp31800 and Pp12633 were unable to grow at 37°C, and the Ps strain was proliferative at 42°C. In contrast, adult sheep serum did not support the growth of any *Pseudomonas* strain tested (data not shown). Fetal bovine serum supported growth of all Pa strains from 28°C to 37°C, and PAO1 and Ps were able to grow at all temperatures. Strains other than Pa and Ps did not proliferate at 37°C or 42°C.

**Table 2:** Optical density of *Pseudomonas* cultures in various media measured at 24 h.

Pseudomonas Strain	Trypticase Soy Broth				100% Fetal Bovine Serum			
	28°C	32°C	37°C	42°C	28°C	32°C	37°C	42°C
**PAO1**	1.28±0.08	1.91±0.03	1.58±0.06	0.52±0.03	0.96±0.05	1.48±0.02	1.47±0.05	0.39±0.01
**Pa10752**	0.41±0.21	0.53±0.00	0.84±0.05	0.00±0.01	0.82±0.10	1.44±0.02	1.18±0.13	0.01±0.02
**Pa31480**	1.28±0.10	1.20±0.10	1.48±0.51	0.58±0.05	0.41±0.59	0.66±0.59	0.75±0.09	0.01±0.00
**Pa700370**	1.44±0.02	1.40±0.11	1.33±0.32	0.76±0.03	1.01±0.48	1.05±0.32	1.16±0.00	0.07±0.04
**Pa700371**	1.43±0.25	1.59±0.15	1.68±0.21	0.66±0.03	1.05±0.00	1.15±0.03	1.06±0.03	0.03±0.00
**Pf13525**	1.53±0.02	0.81±0.57	0.00±0.01	0.00±0.01	0.59±0.26	0.05±0.22	0.00±0.00	0.01±0.01
**Pp12633**	0.96±0.00	1.56±0.46	0.00±0.00	0.00±0.00	1.11±0.00	1.17±0.58	0.00±0.01	0.00±0.00
**Pp31483**	1.38±0.76	1.48±0.12	0.69±0.07	0.00±0.00	1.05±0.05	0.92±0.04	0.09±0.09	0.01±0.00
**Pp31800**	0.54±0.05	0.38±0.00	0.00±0.02	0.00±0.01	0.55±0.01	0.36±0.05	0.00±0.01	0.00±0.00
**Pp700369**	1.56±0.06	1.65±0.04	0.59±0.01	0.00±0.00	1.15±0.00	1.16±0.02	0.33±0.00	0.00±0.00
**Ps17587**	0.25±0.09	0.32±0.03	0.45±0.13	0.12±0.08	0.36±0.10	0.47±0.28	0.44±0.35	0.28±0.02

Key: Positive for Growth (OD>0.1); Delayed Growth (>12h); Negligible Growth (OD<0.1)

The detection of toxins typically observed in Pa was measured in various media (Table 3). The ability of growing bacteria to lyse red blood cells was tested with sheep blood agar at two different temperatures. Almost all tested strains formed colonies on the blood agar, but Pf13525 did not grow at 37°C. Some Pa strains demonstrated discolouration of the blood agar (α-haemolysis), but no complete haemolysis (β-haemolysis) was observed, and the other *Pseudomonas* strains were not haemolytic either (γ-haemolysis).

**Table 3:** Pseudomonas toxin production.

Strain	Sheep Blood Hemolysis (24 h)		Positive Growth Condition (Incubation Time / Temperature / Media)	
	28°C	37°C	Pyocyanin (Blue-green)	Pyoverdine (Fluorescence)
**PAO1**	γ	γ	7 d / RT^1^ / TSB^2^	48h / 37°C / MacConkey, Nutrient Broth, Pseudomonas Selective, Starch, TSB, Urea
**Pa10752**	γ	γ	Not observed	48h / 37°C / MacConkey, Nutrient Agar, Pseudomonas Selective, Starch, TSB, Urea
**Pa31480**	γ	α	48 h / 37°C / TSB	48h / 37°C / Starch, Urea
**Pa700370**	α	α	7 d / RT / TSB	48h / 28°C / Citrate, MacConkey, Starch, TSB, Urea
**Pa700371**	α	α	7 d / RT / TSB	Not observed
**Pf13525**	γ	No growth	Not observed	48h / 28°C / TSB, Urea
**Pp12633**	γ	γ	Not observed	24h / 28°C / Nutrient Agar, Urea
**Pp31483**	γ	γ	Not observed	48h / 28°C / Lysine-Iron, MacConkey, Starch, TSB, Urea
**Pp31800**	γ	γ	Not observed	48h / 28°C / Nutrient Agar
**Pp700369**	γ	γ	Not observed	48h / 28°C / MacConkey, Nutrient Agar
**Ps17587**	γ	γ	Not observed	Not observed

^1^ Room Temperature

^2^ Trypticase Soy Broth

Pyocyanin is a characteristically blue-green pigment that is easily identifiable on agar plates. Only Pa strains demonstrated a blue-green pigment. The exception was Pa10752, which failed to generate pigmentation at any growth condition observed. The other *Pseudomonas* strains did not show any coloration of any tested agar.

The presence of the fluorescent siderophore, pyoverdine, was also investigated. All strains but two (Ps and Pa700371) produced a fluorescent pigment, although the conditions for production varied considerably.

### Antimicrobial Susceptibility

The ability of the *Pseudomonas* species to grow in the presence of various antibiotics was tested using a liquid dilution assay (Tables 4, 5 and 6). The minimum inhibitory concentration (MIC) values were used to determine whether the strains were susceptible, intermediate, or resistant to antibiotics according to EUCAST MIC breakpoints (Table 4). A scoring system, termed the SIR score, was assigned for each bacterium and antibiotic, based on whether the bacterium was susceptible (value of 0), intermediate (value of 1) or resistant (value of 2). Those bacteria with the greatest total SIR score were considered the most resistant to the antibiotics tested. This analysis showed that most Pa strains were relatively susceptible, while Pf13525 demonstrated greatest resistance (Table 5). The reference strain, PAO1, was intermediate in SIR ranking. When the antibiotics were compared for their relative effectiveness against the various strains, ciprofloxacin, colistin, and gentamicin demonstrated most effectiveness, and aztreonam (five resistant strains) and doxycycline (four resistant strains) were relatively ineffective (Table 6).

**Table 4:** Antibiotic susceptibility of *Pseudomonas* strains used in this study.

Antibiotic Minimal Inhibitory Concentration (MIC) (ug/mL +/- standard deviation)							
	Aztreonam	Ceftazidime	Ciprofloxicin	Colistin	Doxycycline	Gentamicin	Meropenem
**S/I/R** ^**1**^	**1/2-16/16**	**8**	**0.5/1/1**	**4**	**0.5/1/2**	**4**	**2/4-8/8**
**PA01**	6.0+/-0	1.6+/-0.7	0.4+/-0.0	1.3+/-0.9	9.0+/-3.5	4.2+/-4.4	0.8+/-0.0
**Pa10752**	7.8+/-4.0	3.6+/-2.1	0.4+/-0.1	1.2+/-1.0	10.8+/-2.7	2.1+/-0.8	0.4+/-0.0
**Pa31480**	16.0+/-6.9	13.5+/-7.5	2.2+/-2.6	3.4+/-1.9	16.0+/-6.9	1.0+/-0.4	1.5+/-1.1
**Pa700370**	6.0+/-0.0	5.6+/-1.1	0.5+/-0.2	1.8+/-0.9	6.0+/-0.0	1.0+/-0.4	3.7+/-4.5
**Pa700371**	0.9+/-0.6	1.7+/-0.9	0.4+/-0.0	0.5+/-0.2	0.4+/-0.0	0.6+/-0.2	0.5+/-0.2
**Pf13525**	>24	20.4+/-8.0	0.4+/-0.0	14.4+/-5.4	0.8+/-0.4	2.3+/-2.3	>24
**Pp12633**	18.0+/-8.5	7.2+/-2.7	0.4+/-0.0	0.5+/-0.2	1.0+/-0.4	0.4+/-0.0	7.2+/-2.7
**Pp31483**	>24	4.7+/-4.3	0.4+/-0.2	1.7+/-1.4	4.7+/-5.0	6.8+/-11.5	1.5+/-1.2
**Pp31800**	>24	4.0+/-2.3	0.4+/-0.2	0.5+/-0.2	10.4+/-22.1	1.4+/-0.3	0.5+/-0.2
**Pp700369**	>24	9.9+/-10.3	0.4+/-0.0	2.2+/-4.3	3.1+/-2.3	1.1+/-1.0	9.4+/-7.4
**Ps17587**	6.6+/-3.8	19+/-8.0	0.4+/-0.0	0.4+/-0.0	0.7+/-0.2	2.4+/-0.8	1.5+/-1.3

**Table 5:** SIR Ranking of *Pseudomonas* strains used in this study.

Ranked SIR Score	Strain
0	Pa700371
3	Pa10752
3	Pa700370
4	Pp12633
4	Ps17587
5	PA01
5	Pp31800
6	Pp31483
7	Pa31480
8	Pp700369
9	Pf13525

**Table 6:** SIR Ranking of Antibiotics used in this study.

Ranked SIR Score	Antibiotic
2	Ciprofloxicin
2	Colistin
4	Gentamicin
6	Meropenem
8	Ceftazidime
15	Aztreonam
15	Doxycyline

^1^S/I/R values correspond to antibiotic susceptible, intermediate and resistant breakpoints as provided by EUCAST (www.bsac.org.uk). Single values represent breakpoints for antibiotic resistance. EUCAST data have been produced in part under ECDC service contracts and made available at no cost by EUCAST and can be accessed freely on the EUCAST website www.eucast.org.

### Bacteria-induced Cytotoxicity

**Fig 1:**
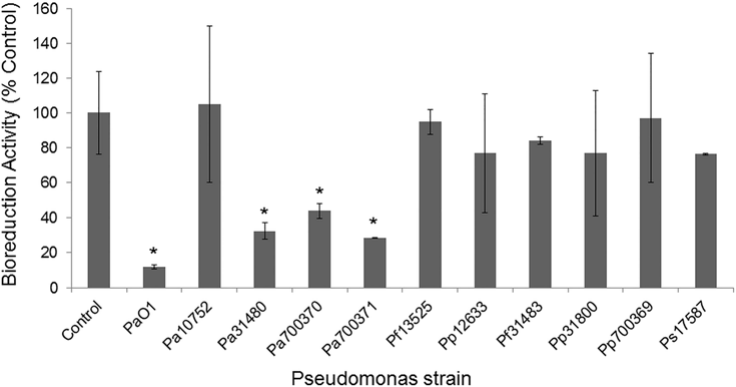
Cellular Viability During *Pseudomonas* Exposure. HT29 cells were exposed to *Pseudomonas* strains for 24 h at 37°C. Bacteria were rinsed from the wells, and one mg mL^-1^ MTT was added. MTT formazan generated after two h incubation was solubilized with DMSO and quantified by absorbance at 505 nm with a multiwell spectrophotometer. Data represent the mean bioreduction activities of three wells ± standard deviation compared to saline-treated wells. Asterisks indicate statistically different values compared to control exposures as determined using ANOVA and Dunnett’s Multiple Comparison Test (p < 0.05).

In order to determine whether the bacteria could cause damage to cultured cells *in vitro*, exposure assays were conducted with the colonic epithelial cell line, HT29. Twenty-four hour exposures were followed by a measurement of cellular metabolic activity (bioreduction activity) as measured with the MTT assay. As shown in Fig 1, the Pa strains (except Pa10752) demonstrated most toxicity (15 to 40% of control cell bioreduction). Observation of the exposure wells by microscopy revealed that the loss in MTT formazan was due to both the loss of cell adhesion and formazan production. Exposure to either Pa10752 or the other *Pseudomonas* strains resulted in non-statistically significant changes in bioreduction ranging from 77 to 98% of control.

### Early Murine Responses Following Bacterial Exposure

**Fig 2:**
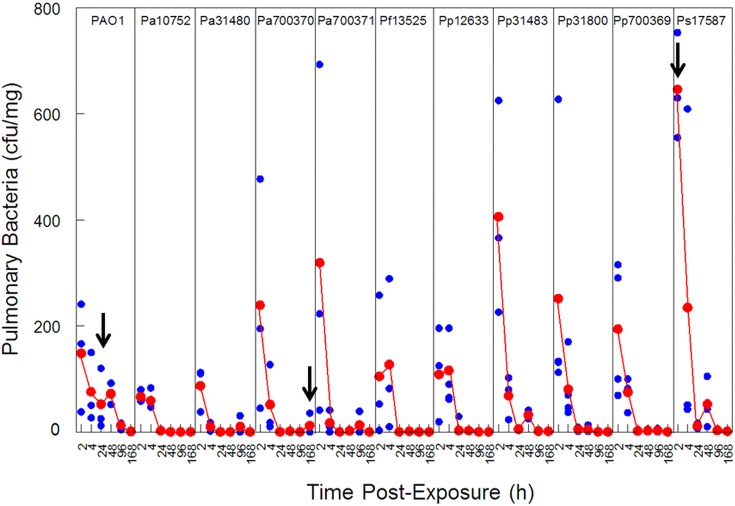
Pulmonary Clearance of *Pseudomonas* Strains. At various times following endotracheal exposure of 10^6^ cfu per mouse of each *Pseudomonas* strain, the lungs of mice were harvested, homogenized and serially diluted in physiological saline. Dilutions were spread-plated onto nutrient broth and bacteria were enumerated 18 h later. The red markers represent the mean colony count; blue markers represent counts from each mouse. Black arrows point to noteworthy features described in the Results section.

Following endotracheal exposure, the number of bacteria in lungs was enumerated at intervals to determine the rate of bacterial clearance. No bacteria were recovered from mice treated with saline alone. Fig 2 shows pulmonary bacteria levels from two to 168 h post-exposure. Most rapid clearance was observed between zero and 24 h post-exposure. At two hours post-exposure, Ps17587 showed marginally greater levels than the other bacteria, but numbers dropped rapidly thereafter. Almost all bacteria were completely eliminated at the 168 h time-point, with the exception of Pa700370. At 168 h post-exposure, this strain resulted in one of three mice with residual bacteria (not observed at 24, 48, or 96 h), albeit at low levels. Notably, mice exposed to PAO1 showed bacteria persisting between four and 48 h; this was not observed with any of the other strains.

**Fig 3:**
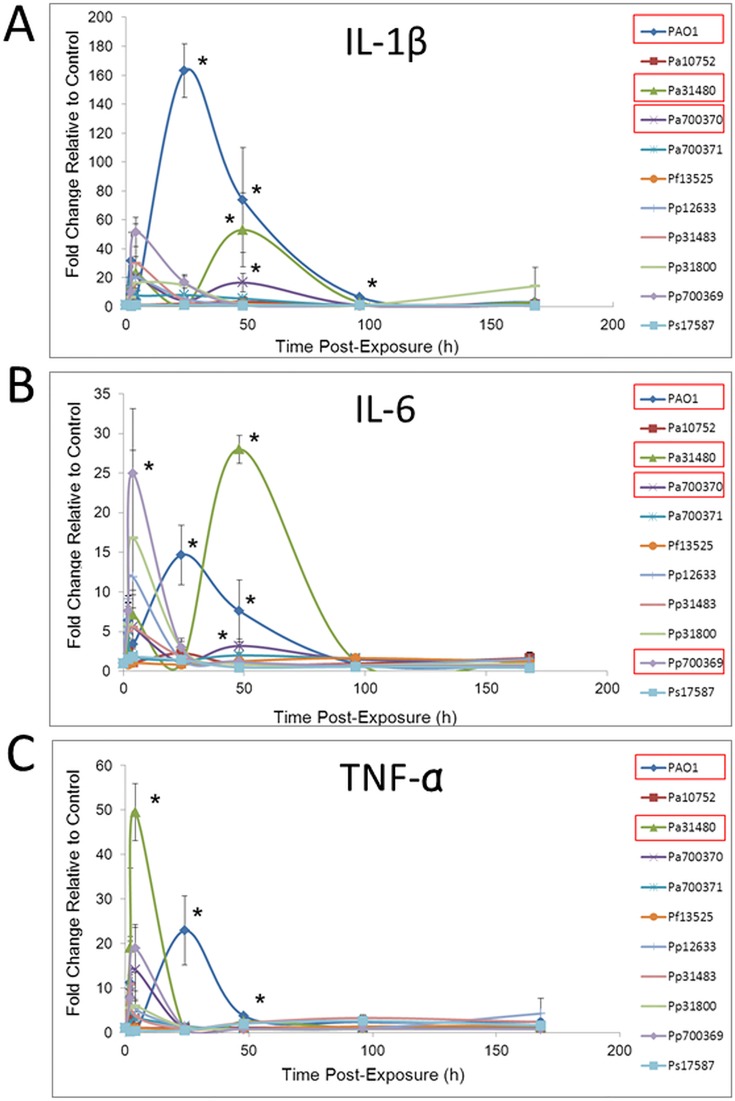
Pulmonary Cytokine Levels During Pseudomonas Exposure. Balb/c mice were endotracheally instilled with saline or 10^6^ cfu of each *Pseudomonas* strain. At various times following exposure, animals were euthanized and lungs were harvested. Following tissue homogenization, cytokine levels were measured using a multiplex bead array system. Data points represent the mean of three or four treated mice. Asterisks indicate statistically different values compared to saline exposures, as determined using ANOVA and Dunnett’s Multiple Comparison Test (*p* < 0.05). Bacteria with significant differences are indicated with red boxes in the graph legends.

Portions of lung were homogenized and analysed for levels of the pro-inflammatory cytokines, interleulin (IL) -1β, IL-6 and tumour necrosis factor (TNF)-α. Fig 3 demonstrates that some, but not all Pa strains were able to elevate cytokine levels. PAO1 and Pa31480 induced the greatest increase in all pro-inflammatory cytokines examined (up to 163-fold for PAO1-stimulated IL-1β). Pa700370 caused elevated levels of IL-1β and IL-6, as well as a statistically non-significant increase in TNF-α (14-fold). Pa10752 and Pa700371 did not cause any significant elevations in pro-inflammatory cytokines. Other than Pp700369, which resulted in a 25-fold mean increase in IL-6 relative to control, no other *Pseudomonas* strain caused significant elevations in any pro-inflammatory marker observed. For all strains, cytokine levels resumed to background control values at one week post-exposure.

**Fig 4:**
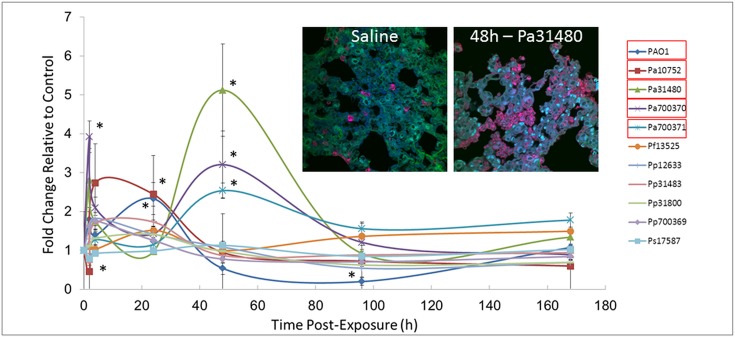
Pulmonary Granulocyte Infiltration during Pseudomonas Exposure. Balb/c mice were endotracheally instilled with saline or 10^6^ cfu of each *Pseudomonas* strain. At various times following exposure, animals were euthanized and lungs were harvested. Lungs were sectioned and stained with fluorescently tagged Ly6G antibody (inset shows example micrographs). Numbers of positively stained cells (red) were enumerated from nine micrograph fields (three fields from three mice for each treatment) and expressed as fold-change compared to those from saline-exposed mice. Asterisks indicate statistically different values compared to saline exposures, as determined using ANOVA and Dunnett’s Multiple Comparison Test (*p* < 0.05). Bacteria with significant differences of at least 2-fold are indicated with red boxes in the graph legends.

Certain *Pseudomonas* strains clearly induced a strong pro-inflammatory response, so pulmonary inflammation was monitored following exposure. This was done by enumerating granulocytes in lung tissue from micrographs such as those shown in insets of Fig 4. All Pa strains, but no other species were able to elevate numbers of pulmonary granulocytes between two and 48 h, and levels stabilized to control values by one week (Fig 4). The response appeared to be separated into two phases; an initial activation that occurred between two and 24 h (PAO1, Pa10752, Pa700370), and a delayed response at 48 h post-exposure (Pa31480, Pa700370, Pa700371). Furthermore, there was statistically significant drop in granulocyte levels at two hours for Pa10752 (2-fold) and 4 d for PAO1 (5-fold) exposure. No other *Pseudomonas* strain caused a significant reduction in levels of granulocytes.

To determine if exposures were able to modify levels of blood cells a haematology analyser was used to monitor leukocyte differentials. Generally, the data trends were difficult to interpret. No discernible patterns were observed for a predominant leukocyte population that was activated or suppressed, which *Pseudomonas* strain caused changes, or what time intervals were observed to be significant (data not shown).

**Fig 5:**
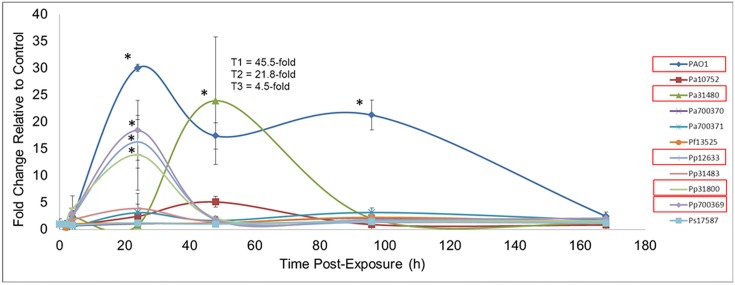
Serum Amyloid A during Pseudomonas Exposure. Balb/c mice were endotracheally instilled with saline or 10^6^ cfu of each *Pseudomonas* strain. At various times following exposure, blood was collected by cardiac puncture. Blood was processed for SAA detection by ELISA. Data is expressed as the fold-change compared to control values ± relative error (n = 3). For Pa31480 treatment at 48 h, the SAA values for each mouse are indicated by T1, T2, and T3. The variation in these values explains the large relative error observed. Asterisks indicate statistically different values compared to saline exposures, as determined using ANOVA and Dunnett’s Multiple Comparison Test (*p* < 0.05). Bacteria with significant differences are indicated with red boxes in the graph legends.

Another measure of systemic response to *Pseudomonas* exposure was that of blood SAA (Fig 5). At 24 h post-exposure, several Pp strains induced elevated SAA levels. At this time-point, PAO1 induced the largest increase (30-fold). Pa31480 did not cause changes at 24 h, but induced a mean 24-fold increase at 48 h. This mean result had a high relative error due to high variation among replicates within the treatment group. By 96 h, only PAO1 exposures demonstrated sustained elevation of SAA (21-fold), and by one-week post-exposure, levels for all treatments had resumed to control values.

## Discussion

This paper investigates several functional virulence endpoints to predict which Pseudomonas species or strains could be safer candidates for biotechnology applications. Table 7 summarises the methodologies that were useful for discriminating virulence of the *Pseudomonas* species tested. These methodologies focus on growth of the organism in mammalian environments, cytotoxicity and immunological responses of mice following exposure. Although *Pseudomonas* spp. possess a number of well-defined virulence factors [24,34], we intentionally selected tests that were primarily based on activity or effects, as opposed to the presence or absence of a gene or protein product. One reason for this decision was that by developing a microorganism-independent regime, it could potentially be applied to other microorganisms that have less well-defined virulence factors, and hence be used to establish information on microorganisms for which there is a paucity of data. Secondly, there is substantial variability in the expression of specific virulence determinants in laboratory and clinical strains [35], and their involvement in pathogenesis remains unclear.

**Table 7:** Summary of Assays for Comparing Potential Virulence Characteristics of *Pseudomonas*.

*Pseudomonas* Strain	Growth at 37°Cin TSB	Growth at 42°Cin TSB	Pyocyanin (Blue-green Pigment)	HT29 Cytotoxicity (24 hrs)	Pulmonary IL-1beta	Pulmonary IL-6	Pulmonary TNF-alpha	Pulmonary Neutrophil Infiltration	Serum Amyloid A (≥ 48 hrs)
**PAO1**	+	(+)	+	+	+	+	+	+	+
**Pa10752**	+	-	-	-	-	-	-	+	-
**Pa31480**	+	(+)	+	+	+	+	+	+	+
**Pa700370**	+	(+)	+	+	+	+	-	+	-
**Pa700371**	+	(+)	+	+	-	-	-	+	-
**Pf13525**	-	-	-	-	-	-	-	-	-
**Pp12633**	-	-	-	-	-	-	-	-	-
**Pp31483**	+	-	-	-	-	-	-	-	-
**Pp31800**	-	-	-	-	-	-	-	-	-
**Pp700369**	(+)	-	-	-	-	+	-	-	-
**Ps17587**	+	(+)	-	-	-	-	-	-	-

Key:—no response, (+) partial response, + positive response

A wide growth temperature range is a commonly considered as an advantageous trait for pathogenesis, and growth at 37°C and 42°C has been associated with pathogenic phenotypes [36–38]. Strains unable to proliferate at higher temperatures would be less likely to be active at human body temperatures. Furthermore, adult sheep serum was not permissive for growth of any strain, potentially because of the presence of complement components. In contrast, FBS, which contains much less complement, permitted the growth of all Pa isolates at 37°C. These proliferation experiments demonstrated that the Pa strains were most able to grow under the widest temperature range and in mammalian conditions.

Besides the ability to proliferate, microorganisms generate substances that can help to evade or inhibit host defence responses and provide nutrients necessary to sustain growth. Several observations were made during routine nutrient-agar growth tests that provided insight into the presence or absence of specific virulence determinants. None of the *Pseudomonas* strains examined were able to lyse red blood cells, a common strategy among bacteria to obtain iron [39]. Almost all Pa strains were capable of discolouring the media around the colonies (Table 3) due to pyocyanin production. Pyocyanin is a phenazine-based compound that induces oxidative stress through the generation of reactive oxygen species [40]. Its production is under the control of the *Pseudomonas* quorum sensing system [41]. Pyocyanin contributes to virulence by affecting electron transport, cellular respiration, energy metabolism, and innate immune responses [32]. The presence of pyocyanin suggests that producing strains are capable of causing toxicity; it does not exclude the possibility that non-producing strains are pathogenic using other mechanisms.

Another substance produced by *Pseudomonas* species is pyoverdine, which is a fluorescent siderophore [42]. Data presented in Table 3 shows that almost all *Pseudomonas* strains tested (except Ps17587 and Pa700371) produced a fluorescent substance indicative of pyoverdine production. The fluorescence observed may represent the relatively iron-deficient media, which is a trigger for pyoverdine production [31]. These results suggest that pyoverdine is not a discriminatory indicator of virulence status of *Pseudomonas* species.

Antibiotic MIC tests enabled a ranking of the most resistant strains (Pf13525 –industrial; Pp700369—environmental), and the most ineffective antibiotics (aztreonam, doxycycline) (Tables 4, 5 and 6). Several recent publications have demonstrated that *Pseudomonas* species other than Pa that were isolated from environmental sources can be highly resistant to currently used antibiotics [43], especially those from hydrocarbon-contaminated sites [44]. Yet in another study, 60% of strains isolated from hospital settings demonstrated multi-drug resistance compared to 12% ‘outdoor’ isolates [45]. Although MIC tests were not useful for virulence determination, this data highlights the importance of frequent monitoring of the efficacy of individual antibiotics. Furthermore, antimicrobial tests should be a critical component in virulence testing, since Pa strains cannot be assumed to be the most problematic.

The toxicity of the strains was evaluated using an epithelial cell line exposure assay, as well as a murine endotracheal exposure model, both of which were previously used by us to predict the toxicity of *Bacillus* and *Acinetobacter* species [21,29,46]. In the animal model, PAO1 was cleared most slowly, with a small residual content as late as 48 h post-exposure, which may have been sufficient to result in a more aggressive immunological response observed for PAO1 compared to the other strains. Clearance was sometimes erratic (PAO1, Pp31483, Ps17587), which may suggest, colonization, adherence to airways.

A study that monitored clearance of a cystic fibrosis Pa isolate, TBCF10839, in C57BL/6JZtm mice, also demonstrated slow clearance for the first four hours, but rapid decline to negligible levels at 24 h post-exposure [47]. This slow initial (4–8 h) clearance may be typical of a virulent isolate, but differs from our PAO1 result in that we did not observe complete clearance until one week post-exposure. In another study, with intratracheal exposure to 10^7^ cfu of PAO1, levels were ~10^4^ cfu/mouse at 4d, and 2 x 10^5^ cfu/mouse at 7d [48]. A dose of just 2-fold higher resulted in 88% mortality of the mice. These results corroborate our observation that clearance rate may be variable and dependent on multiple assay parameters. Nevertheless, the low levels of bacteria present even after just two hours of exposure, and the subtle differences to that of other *Pseudomonas* species tested, suggest that pulmonary clearance may not be the most informative indicator of *Pseudomonas* virulence.

The presence of the bacteria in the lungs was expected to induce a pro-inflammatory response. Pulmonary pro-inflammatory cytokines IL-1β, IL-6 and TNF-α measured in lungs (Fig 3) were relatively high during exposure to Pa strains, especially PAO1 and Pa31480. In contrast, Pa10752 did not cause statistically elevated levels of any cytokine tested. Our results demonstrate the prognostic value of pro-inflammatory cytokines.

Pulmonary neutrophil levels were monitored since this is a primary mechanism for Pa inactivation or removal [34]. Fig 4 demonstrated that only Pa strains resulted in the infiltration of neutrophils. Similar to our results, Xu and colleagues observed maximal induction of neutrophils in brochioalveolar lavage fluid at 48 h [49]. The neutrophil response during infection by Pa is usually strong [35]. Whether neutrophils were involved in clearance of the non-Pa species is not known, but neutrophil levels in mice exposed to these species were statistically unchanged compared to controls. Macrophage-mediated clearance may have been involved in the reduction of these species even though depletion of macrophages in mice prior to PAO1 exposure did not alter the pulmonary clearance rate [50].

The acute phase response protein, SAA, is generated by hepatocytes during inflammation, and is primarily induced by IL-1β, IL-6 and TNF-α [51,52]. Typically, SAA levels rise three to six hours after onset of inflammation and resume to background levels at four days [53]. Our data demonstrated that for most species, the levels of SAA were maximal at 24 h and resumed to normal by 48 h. However, PAO1 and Pa31480 demonstrated a distinctly different pattern in that SAA levels from exposure were elevated at 48 h. Levels for PAO1 remained high (20-fold) even at 4 days, and only diminished to basal levels at the next sampling point of one week post-exposure. SAA has several functions during the immune response [51]. It can function as a pro-inflammatory cytokine, promoting production of IL-1β, IL-6, IL-8, granulocyte macrophage-colony stimulating factor (GM-CSF), and function as a neutrophil and macrophage chemoattractant. It has been demonstrated to interact with several receptors that act to amplify the innate immune response (TLR2, TLR4, FPRL-1, scavenger receptors, RAGE). The high rate of SAA synthesis, with a turnover of about 24 h, coupled with the late and sustained increase in SAA levels, suggests that PAO1 and Pa31480 were recognized systemically as the more virulent strains.

## Conclusions

Several assays, summarized in Table 7, were used for discriminating the virulence of the *Pseudomonas* species. This data showed that non-Pa strains were relatively avirulent. Of the Pa strains, Pa31480 showed virulence characteristics similar to that of PAO1, and should be examined more critically for its pathogenic potential, since it is an industrial strain. In contrast, Pa10752, which was isolated from a clinical source, and is not a known biotechnology strain was the only Pa strain that did not proliferate at 42°C, produce pyocyanin-like media colouration, induce any significant cytotoxicity, or cause negative effects in our transient infection mouse model. As such, this strain may be relatively safe to adopt for biotechnology applications, especially if it possesses beneficial characteristics that are not available in other industrial *Pseudomonas* strains. Further genetic analyses are recommended to confirm if specific determinants important for virulence were suppressed under the exposure conditions tested. We conclude that several tests are necessary for revealing pathogenicity potential, and that it is critically important to avoid reliance on taxonomical designations or isolation source for prediction of microbial hazard during selection of strains for biotechnology applications.
